# Investigation of metabolites accumulation in medical plant *Gentiana rigescens* during different growing stage using LC-MS/MS and FT-IR

**DOI:** 10.1186/s40529-015-0094-6

**Published:** 2015-05-27

**Authors:** Yu Pan, Ji Zhang, Yan-Li Zhao, Yuan-Zhong Wang, Heng-Yu Huang

**Affiliations:** 1 College of Traditional Chinese Medicine, Yunnan University of Traditional Chinese Medicine, Kunming, China; 2 Institute of Medicinal Plants, Yunnan Academy of Agricultural Sciences, Kunming, China

**Keywords:** Gentiana rigescens, Tissue culture, Metabolites variation, LC-MS/MS, FT-IR

## Abstract

**Background:**

*Gentiana rigescens*, an important medicinal plant in China, has been widely cultivated in Yunnan province, China. Previous studies were focused on analysis and determination of the metabolites isolated from this species, the accumulation of these metabolites during growth period are not yet clear. In this study, samples for the experiments were obtained by tissue culture. FT-IR and LC-MS/MS method were performed to distinguish the variation on the major metabolites in *G. rigescens* during growing stage when combined with chemometrics.

**Results:**

Methodology validations were all within the required limits. The metabolites were visually different in tissue culture samples and mature plants. The diversity of metabolites increased proportionally with plant growth. The quantitative analysis showed the content of gentiopicroside was significantly vary during different growing stage. The highest content of gentiopicroside (122.93 ± 7.01 mg/g) was detected in leaf of regenerated plantlet, whereas its content in root significantly increased along with underground parts growth. Moreover, flavonoids mainly distributed in aerial parts showed potential competitive relationship during plant growth.

**Conclusion:**

The distribution and accumulation of metabolites are associated with different parts and plant growth, which provide potential evidences for the rational application and exploitation of *G. rigescens*.

**Electronic supplementary material:**

The online version of this article (doi:10.1186/s40529-015-0094-6) contains supplementary material, which is available to authorized users.

## Background

*Gentiana rigescens* Franch belonging to Gentianaceae is an important medicinal plant in China for treatment of hepatitis, jaundice and dysentery. The root and rhizome of this plant has been officially documented in Chinese Pharmacopoeia as one of raw materials of Gentianae Radix et Rhizoma (Longdan in Chinese), a hepatoprotective agent (State Pharmacopoeia Commission [2010]). According to ethnobotanical information, its aerial parts are also used as folk medicine for treatment of fever and rheumatic arthritis or as antiophidica, when prepared with vegetable oil. The phytochemistry and bioactivities of *G. rigescens* have been intensively studied. More than 100 secondary metabolites with different activities like hepatoprotective, anti-inflammatory, antioxidant and neuritogenic growth have been isolated from this plant (Gao et al. [2010a], [2010b]; Xu et al. [2006], [2007], [2009a]; Wang et al. [2012]). Among them, iridoid glycosides is the most abundant components especially gentiopicroside which content is more than 4.5 % and serve as major active ingredient and standard for quality control (Jiang et al. [2005]; Pan et al. [2015a]; Wang et al. [2012]).

As one of well-known traditional Chinese medicine with remarkable medicinal functions, the wild resources have been under heavy threat owing to human activities and environmental pollution. Although *G. rigescens* have been extensively planted in Yunnan, some disadvantages on cultivation such as continuous cropping obstacle, time-consuming and laborintensive, etc. result in decline of production and quality. Fortunately, plant tissue culture is conducive to the accumulation of biomass and metabolites, in particular with individual metabolites, which amount is multifold higher than control group when treated with appropriate elicitors (Chuang et al. [2014]; Huang et al. [2014]; Kuzovkina et al. [2014]; Kumari et al. [2015]; Marsh et al. [2014]; Su et al. [2014]). However, the efficacy of medical plant, to a large extend, are attributed to synergistic effect of a number of metabolites. The amount of individual metabolites is significantly increased, which might lead to different pharmacological activities and therapeutic effects.

On the other hand, previous studies (Jiang et al. [2005]; Pan et al. [2014], [2015a]; Wang et al. [2012]) on metabolites of *G. rigescens* were most focused on quality assessment, phytochemistry, pharmacology, etc. However, metabolites are not only the efficacious properties for maintaining human health, but also play an important role for resistance to abiotic and biotic threats during plant growth (Hall et al. [2008]; Wink [2003]). To our best knowledge, the accumulation and variation of metabolites in *G. rigescens* have not yet clear.

Currently, analysis of metabolites based on separation technologies such as gas chromatography–mass spectrometry (Hu et al. [2014]), liquid chromatography coupled with photodiode array detector (Yu et al. [2014]), mass spectrometry detector (Won et al. [2014]) and nuclear magnetic resonance spectroscopy (Hilbert et al. [2015]) can rapidly provide complex chemical information and clarify the similarities and differences of bio-samples when combined with chemometrics. However, comprehensive chemical information on metabolites cannot be analyzed in a single chromatogram. Fourier transform infrared spectroscopy (FT-IR) enables to rapid reflect holistic molecular structure-analyte relationships, which is considered as a well-established and non-destructive method for analysis of bio-sample, whereas it fails to recognise the variation of specific compound in sample due to the limited specificity and sensitivity (Karoui et al. [2010]; Lohumi et al. [2014]; Zhao et al. [2014]).

In this study, the variation on distribution and accumulation of metabolites in *G. rigescens* are investigated based on plant tissue culture. Individual parts of sample during different growing stage are subjected to targeted and non-targeted analysis using FT-IR and liquid chromatography tandem mass spectrometry (LC-MS/MS). Moreover, the biosynthetic pathway of iridoid glycosides is also discussed. The combinative comparison approach can reflect the overall chemical difference during different growing stage, which may provide the useful information for reasonable utilization of resources.

## Methods

### Materials and chemicals

**Fig. 1:**
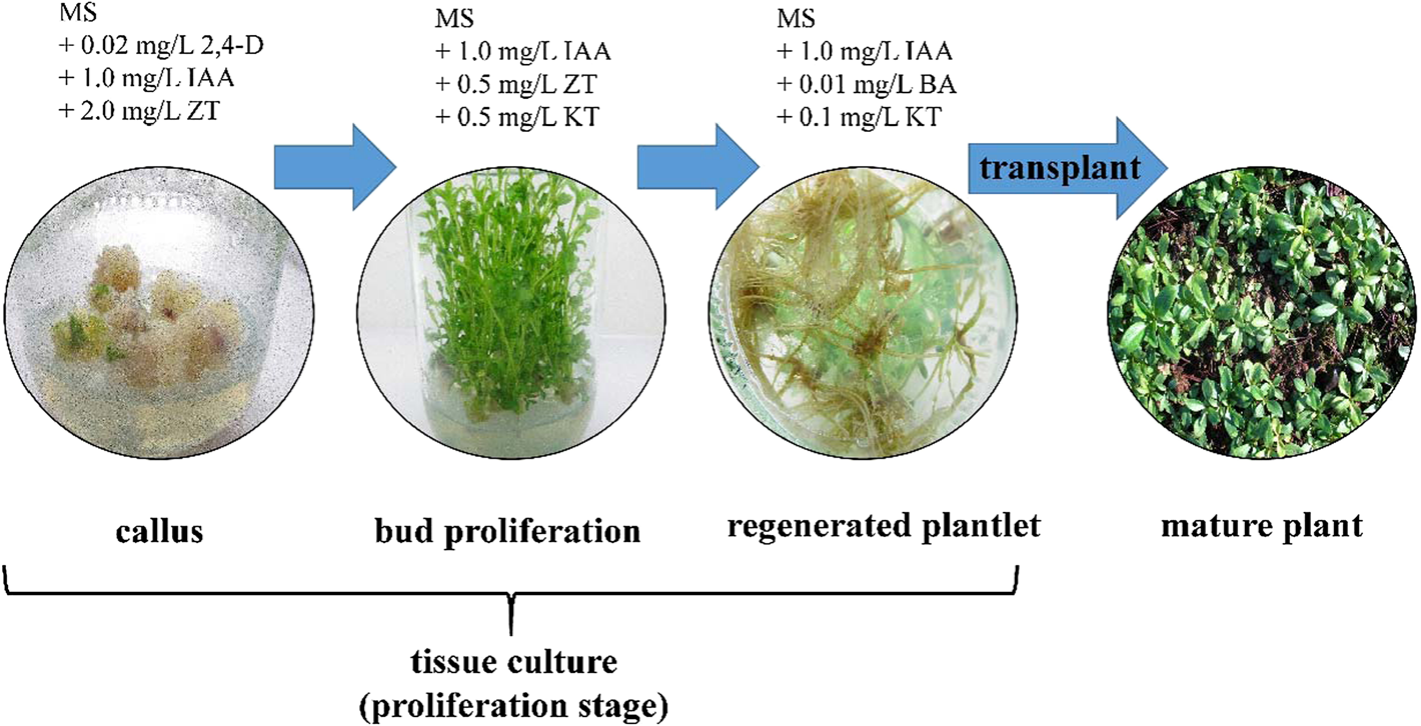
Tissue culture flowchart of *G. rigescens*

Tissue culture materials provide by Dr. Heng-Yu Huang (College of Traditional Chinese Medicine, Yunnan University of Traditional Chinese Medicine) were established using leaves of *G. rigescens.* The operations of tissue culture were completed on a super-clean bench. The plantlets were grown on Murashige-Skoog (MS) medium supplemented different concentration of indoleacetic acid (IAA), zeatin(ZT), dichlorphenoxyacetic acid (2,4-D), kinetin (KT) and 6-benzyladenine (BA) during different stage. The processed of tissue culture is present in Fig. 1. The culturing conditions were incubated at 23 ± 2 °C under cool white fluorescent light at 1500–2000 lx under 10 h per day.

**Fig. 2:**
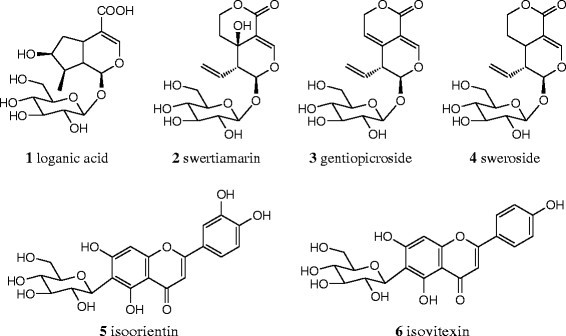
Index compounds of *G. rigescens*. (1. loganic acid; 2. swertiamarin; 3. gentiopicroside; 4. sweroside; 5. isoorientin; 6. isovitexin)

KBr (specpure) was purchased from Tianjin FengChuan Fine Chemical Research Institute (Tianjin, China). Methanol and formic acid (assigned purity > 98 %) were LC grade and purchased from Thermo Fisher Scientific (USA) and Dikmapure (USA), respectively. Water was purified to 18.25 MΩ using Milli-Q system from Millipore (USA). All other chemicals for extraction were analytical grade. The standard compounds (**1**. loganic acid, **2**. swertiamarin, **3**. gentiopicroside, **4**. sweroside, **5**. isoorientin and **6.** isovitexin) (Fig. 2) were provided by Chinese National Institute for the Control of Pharmaceutical and Biological Products (Beijing, China). Their assigned purity were all >98 %.

### Apparatus

Tablet press (YP-2, Shanghai Shanyue instrument Inc) was used to press powder into thin samples. The FT-IR spectrometer (PerkinElmer, USA) equipped with a DTGS detector. IR spectra were recorded from the accumulation of 16 scans in 4000–400 cm^−1^ range with a resolution of 4 cm^−1^.

Separation, quantitation and quantification of metabolites were performed on a Shimadzu Nexera UHPLC tandem mass spectrometry (LCMS-8030, Shimadzu, Japan) equipped with a Shim-pack XR-ODS III (75 × 2.0 mm, 1.6 μm) column, UV detection and triple quadrupole mass spectrometer via an electrospray ionization (ESI) interface. The mobile phase consisted of 0.1 % formic acid in water (A) and methanol (B) was applied at a flow rate of 0.35 mL/min with gradient as follow: initial, 13 % B; 0.31-7.00 min 20 % B linear, 7.01-13.00 min 46 % B linear, 13.01-16.50 min 83 % B linear; followed by a final increase to 90 % in 1 min. After a 3 min wash, the column was reconditioned at 13 % B for 3 min to prepare for the next injection. Injection volume and column temperature were 1 μL and 40 °C, respectively. The detection wavelength was set at 242 nm, where all the standards and UPLC profiling showed a satisfactory performance.

High-resolution electrospray ionization mass spectrometry was performed using a Agilent G6550 QTOF (Agilent technologies Santa Clara, CA, USA) equipped with an ESI inter-face. Mass spectra were acquired in both positive and negative modes over the range *m/z* 100–1000. The capillary voltages were set at 3000 V (positive mode) and 2700 V (negative mode), respectively, and nozzle voltage was 300 V. Sheath gas and drying gas were nitrogen at a flow rate of 3.0 and 14.0 L/min, respectively, nebulizer pressure was 20 psi. The precise molecular mass was determined by the accurate-mass data of the TOF analyzer within a reasonable degree of measurement error, normally with mass errors below 5 ppm in routine analysis, which was sufficient to verify the elemental compositions of the known constituents in *G. rigescens*.

The quantification of the targeted compounds with low concentration was carried out on multiple reaction monitoring (MRM). The settings of MRM were auto-optimized by Labsolutions software (Shimadzu, Japan). The triple quadrupole mass spectrometer parameters were set as follows: nebulizing gas and drying gas were nitrogen at a flow rate of 3.0 and 15.0 L/min, respectively; the interface voltage was set to 4.5 kV; desolvation line (DL) temperature was 250 °C and the heat block temperature was 400 °C. Reference solutions containing ions of *m/z* 503.15 and 1004.60 were continuously introduced into the MS system during the analysis procedure to ensure the accuracy of the measured mass.

### Sample preparation

Fresh samples were dried at 60 °C and ground into fine powder. Then, 2 mg sample was blended with 200 mg KBr powder, ground again and pressed into a tablet. The FT-IR spectra of all samples were collected three times after by subtraction of KBr pellet background.

Sample preparation was based on our previous work (Pan et al. [2015b]). An accurately weighed sample powder (0.1 g) was extracted by ultrasonication with 7 mL 80 % methanol for 35 min. The extract solution were filtered through a paper filter. Then, the filtrate were stored at 4 °C and filtered through a 0.22 μm membrane filter before injection into the LC system for analysis. Injection volume was 1 μL.

### Data analysis

The raw FT-IR spectroscopy were processed by Omnic 8.0 (Thermo Fisher Scientific, USA). The peaks of UV and mass data were picked and filtered by Labsolutions software (Shimadzu, Japan). Principal component analysis (PCA), an unsupervised chemometric approach for classification, was exploited to optimize the complex data set for reflecting relationships among different samples. The PCA was performed by software SIMCA-P^+^10.0 (Umetrics AB, Sweden).

## Results

### Comparative analysis of *G. rigescens* in different growing stage

**Fig. 3:**
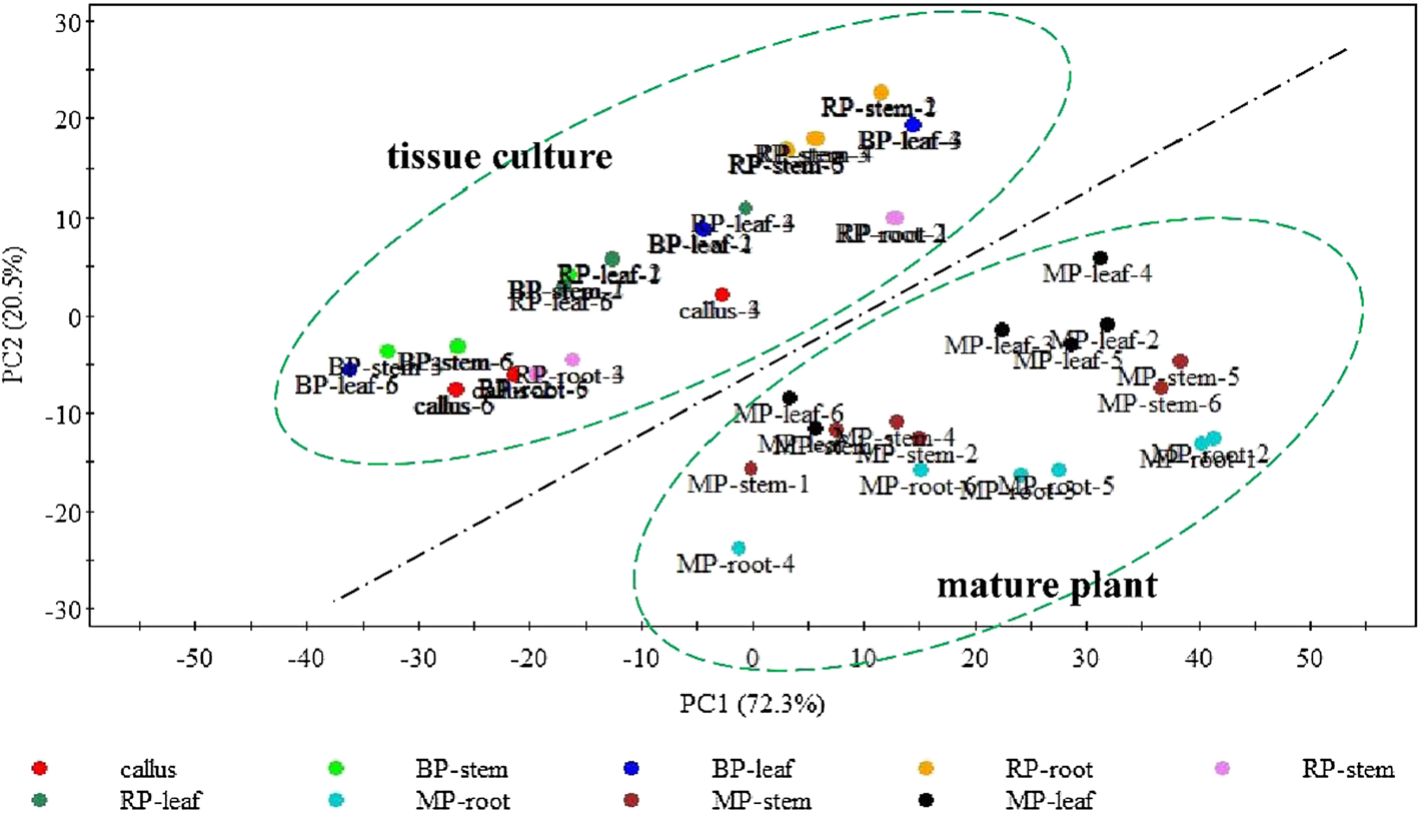
The PCA scores plot of samples during different stage based on FT-IR

FT-IR and LC-MS/MS were used to investigate the metabolites variation in both integrity and detail on chemical information in different plant part and growing stage. There are few visual differences in averaged FT-IR spectra of different sample. In order to explore the relationships between metabolites and plant growth, the 1800–600 cm^−1^ region of the FT-IR spectral data without the interferences of CO_2_ and H_2_O were subjected to PCA. A two-dimension (2D) scores plot (PC1 × PC2) was constructed from a data matrix (623 × 54) by PCA which could visually reflect the similarity between IR spectra and samples in this plot where the closer the points, the more similar the spectral data. In PCA, the first and second principal components cumulatively accounted for 92.8 % of the total variance, which suggested that the former two principal components could explain the proportion of the experimental data. In Fig. 3, sample of mature plant and samples during proliferation stage were explicitly separated into two groups. However, samples in the corresponding groups were crossed and could not be classified according to their plant parts or growing stage. These results implied that the whole metabolome based on FT-IR spectra were significant different, whereas these the detailed variation failed to be monitored especially for content of individual metabolites.

**Fig. 4:**
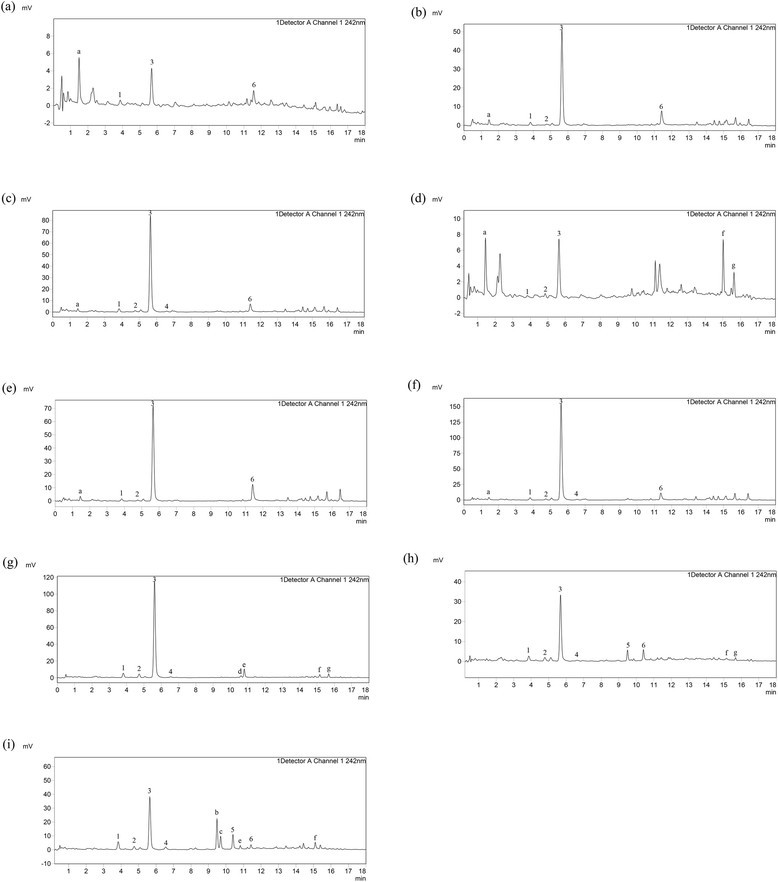
UPLC profiling of *G. rigescens.* (1. loganic acid; 2. swertiamarin; 3. gentiopicroside; 4. sweroside; 5. isoorientin; 6. isovitexin; **a**: callus; **b**: BP-stem; **c**: BP-leaf; **d**: RP-root; **e**: RP-stem; **f**: RP-leaf; **g**: MP-root **h**: MP-stem; **i**: MP-leaf)

Moreover, a targeted method based on LC-MS/MS was designed for monitoring variations of iridoid glycosides and flavonoids. The 80 % MeOH extracts of *G. rigescens* were injected into LC-MS/MS system for analysis. As shown in Fig. 4a-i, peak numbers and their peak areas were visually different in samples, especially for peaks a, 3, 5, 6, b and c, which indicated the accumulation of metabolites varied with plant growth. For example, peak b and c were only found in leaf of mature plant. Furthermore, peak a is one of characteristic marker in chromatogram of callus, whereas it could not detected in chromatogram of mature plant.

Except for peak 1–6 which can be unambiguously identified by standard compounds, other peaks in chromatogram are still unknown. In order to further clarify metabolites variations during growing stage, peak a-g (Additional file 1: Table S1), characteristic markers in chromatogram of samples, were tentatively identified via matching the mass data (high revolution data and MS/MS spectra) with published works on chemical compounds isolated from *G. rigescens*. According to comparison of mass data of standard compounds, peak b and c could be tentatively assigned as flavonoids while peak d-g were iridoid glycosides. In mass spectra of isoorientin and isovitexin (Additional file 1: Figure S1, see supplement data), the characteristic neutral loss of 90 and 120 Da are correspond to *C*-glycosidic structure, which could be considered as diagnosed markers. Interestingly, the mass data of peak b and c (Additional file 1: Figure S1) were highly similar with isoorientin and isovitexin, respectively. Besides, the neutral loss of 162 Da, the feature of glucosyl group, were detected in both peak b and c. Therefore, peak b (*m/z* 593.1513 [M-H]^−^, C_27_H_30_O_15_) and c (*m/z* 611.1503 [M-H]^−^ C_27_H_30_O_16_) were the *O*-glycosidic derivatives of isoorientin and isovitexin, respectively.

Furthermore, a neutral loss of 136 Da which may correspond to the loss of a dihydroxy benzoyl group via a classical McLafferty-type rearrangement (Tan et al. [1996]; Xu et al. [2009b]) was observed in mass spectrum of peak d-g. By further analyzing the mass data, peak d-g were dihydroxy benzoyl iridoid glycosides. Their mass spectrum and fragmentation pattern are shown in Additional file 1: Figure S2. In mass spectrum of peak a, the product ions at *m/z* 153 and 109 were correspond to losing a glucose (162 Da) and carboxyl group (44 Da), indicating peak a (*m/z* 315.0832 [M-H]^−^ C_13_H_16_O_9_) were tentatively assigned as *O-* glucosyl-dihydroxy benzoyl acid (Additional file 1: Figure S3) (Xu et al. [2009a]). All compounds but peak 1–6 were identified by matching corresponding mass data in previous studies.

### Method validation and quantification

Standard solutions of each compounds with seven different concentrations were prepared individually in methanol and were injected into LC-MS/MS system for generation of external standard calibration curves. Calibration curves for each compound were performed by plotting the peak area (y) against the concentrations (x, μg/ml). The correlation coefficients (R^2^) of each calibration curve were more than 0.9992. The limits of detection (LOD) and quantification (LOQ), S/N (signal-to-noise ratio) of 3 and 10, were determined by serial dilution of each standard solution using the described conditions. The LOD and LOQ for UV detector and mass analyzer, together with standard calibration curves of each standards, were listed in Table 1.

**Table 1:** Linear regression and MRM parameters of standards

Analytes	Regression equation	Linearity range (μg/mL)	R^2^	LOD (μg/mL)	LOQ (μg/mL)	Ions pairs	CE (eV)
Loganic acid	y = 2099.0x + 19518.7	50-500	0.9996	0.34	1.01	421 > 375	27
Loganic acid^a^	y = 200150.0x + 2801.4	0.05-5	0.9997	0.004	0.022	421 > 213	22
Swertiamarin	y = 2151.2x + 11429.0	10-150	0.9998	0.27	0.92	419 > 179	18
Swertiamarin^a^	y = 169057.0x + 3753.2	0.1-5	0.9995	0.005	0.027	419 > 141	23
Gentiopicroside	y = 1494.4x + 21722.0	5-1500	0.9995	0.23	1.03	401 > 197	14
-	-	-	-	-	-	401 > 89	21
Sweroside^a^	y = 292679.0x + 5034.6	0.5-10	0.9992	0.008	0.037	359 > 197	−31
-	-	-	-	-	-	359 > 127	−10
Isoorientin	y = 2945.4x-6172.4	5-250	0.9993	0.57	2.33	447 > 327	27
Isoorientin ^a^	y = 278957.0x + 4532.8	0.05-5	0.9994	0.007	0.041	447 > 357	23
Isovitexin	y = 1979.7x + 19518.7	5-250	0.9998	0.63	1.97	431 > 311	24
Isovitexin ^a^	y = 409813.0x + 1523.7	0.05-5	0.9996	0.007	0.037	431 > 331	22

-: not mentioned

^a^: determination by MRM

Precision was evaluated by intra- and inter-day variation which determined by analyzing mixed standard solutions with known concentration six times within a day and on three consecutive days in triplicate. The intra- and inter-day precision of peak area (expressed in terms of %RSD) were in the range of 0.76–2.27 %. Accuracy was validated by recovery test performed by accurately adding three different amounts (low, medium and high spike) of each standard to the crude sample. The recovery rates of six standards were ranged from 97.3-102.6 % and their RSD values were less than 3 %. Then, six independently samples analysed by repeating the described produce of sample preparation under this chromatographic condition were used to investigate the repeatability. These results are displayed in Table 2.

**Table 2:** Precision accuracy and repeatability (%RSD) of this method

Analytes	Intra-day RSD%			Inter-day RSD%	Accuracy (*n* = 9)		Repeatability (*n* = 6)
	Day 1	Day 2	Day 3				
	P_a_	P_a_	P_a_	P_a_	Mean recovery	RSD%	RSD%
UV detector							
Loganic acid	1.13	2.09	2.36	1.58	102.6 %	1.93	1.79
Swertiamarin	1.53	1.72	1.97	1.46	101.8 %	2.62	1.88
Gentiopicroside	1.74	1.85	2.27	1.38	98.7 %	1.77	1.69
Isoorientin	1.22	2.21	2.33	2.25	102.2 %	2.56	1.71
Isovitexin	1.27	1.85	1.76	1.64	97.3 %	2.13	2.07
MRM							
Loganic acid	1.26	1.58	1.71	1.57	99.3 %	1.99	1.86
Swertiamarin	1.45	1.31	1.67	1.78	100.9 %	1.86	1.22
Sweroside	1.48	1.37	1.94	0.76	101.4	1.78	1.85
Isoorientin	0.95	1.08	1.88	1.21	98.7 %	2.14	1.29
Isovitexin	1.66	1.33	1.92	1.59	98.9 %	1.98	2.24

P_a_: peak areas

In this study, the identification of standards in the chromatogram was confirmed by retention times and ion pairs determined by MRM. Quantification depended on the external standard method. The contents of the six standards in *G. rigescens* during different rowing stage are listed in Table 3. The results showed that the content of the six standards differed greatly in different stage. The lowest contents of all standards were found in callus. Gentiopicroside was the highest yield compound in the whole growth stage. The highest gentiopicroside yield was found in leaf of regenerated plantlet (122.93 ± 7.01 mg/g), followed by root of mature plant (96.78 ± 8.54 mg/g). Interestingly, gentiopicroside yield was reduced and tended to accumulate in root after regenerated plantlet. Furthermore, root did not contain the two flavonoids. The highest content of the two flavonoids were observed in leaf. For isoorientin, the highest content was found in mature plants. On the contrary, the highest isovitexin yield was detected in proliferation stage.

**Table 3:** Mean Contents (mg/g) of six standards (*n* = 6)

Analytes	Loganic acid	Swertiamarin	Gentiopicroside	Sweroside	Isoorientin	Isovitexin
Callus	0.053 ± 0.002^a^	0.017 ± 0.001^a^	0.35 ± 0.25	0.05 ± 0.001^a^	0.013 ± 0.002^a^	0.075 ± 0.01^a^
BP-stem	3.12 ± 0.17	1.66 ± 0.22	45.11 ± 2.21	0.06 ± 0.001^a^	0.016 ± 0.002^a^	2.25 ± 0.62
BP-leaf	3.73 ± 0.28	2.78 ± 0.44	85.13 ± 3.93	0.81 ± 0.04^a^	0.021 ± 0.002^a^	2.17 ± 0.58
RP-root	0.034 ± 0.005^a^	0.038 ± 0.005^a^	0.55 ± 0.03	0.03 ± 0.007^a^	-	-
RP-stem	3.66 ± 0.02	2.65 ± 0.02	62.13 ± 5.66	0.36 ± 0.03^a^	0.036 ± 0.001^a^	2.87 ± 0.72
RP-leaf	4.55 ± 0.72	2.83 ± 0.14	122.93 ± 7.01	0.41 ± 0.04^a^	0.047 ± 0.002^a^	3.07 ± 0.33
MP-root	8.52 ± 0.59	6.16 ± 0.72	96.78 ± 8.54	0.27 ± 0.07^a^	-	-
MP-stem	4.90 ± 0.11	3.06 ± 0.25	17.58 ± 3.21	0.46 ± 0.02^a^	1.58 ± 0.32.	0.83 ± 0.02
MP-leaf	5.51 ± 0.21	4.11 ± 0.21	26.05 ± 4.33	1.16 ± 0.13^a^	2.84 ± 0.52	0.85 ± 0.22

^a^: Determination by MRM

## Discussions

According to the results of comparative analysis, the distribution and accumulation of metabolites are associated with plant growth. The whole metabolome in different stage are significantly different. The reason might be attributed to (1) elicitors in medium and (2) environment conditions such as soil, sun exposure time and rainfall, which could also result in fluctuation on the distribution and accumulation of metabolites after transplant (Manukyan [2011]; Marsh et al. [2014]; Xie et al. [2011]). Additionally, the proportion among metabolites vary with plant growth. The proportion of peak a (*O-* glucosyl-dihydroxy benzoyl acid) in chromatogram gradually reduce with the increase of other metabolites. Based on the identification of peak d-g, *O-* glucosyl-dihydroxy benzoyl acid may transformed into dihydroxy benzoyl iridoid glycosides via esterification of iridoid glycosides. The obvious differences are observed in chromatogram of leaf during different stage especially for peak b and c (*O*-glycosidic derivatives of isoorientin and isovitexin) which only is detected in mature plant. These result implied that the synthesis and transform of metabolites with complex molecular structure tend to be in mature plant with more abundant substance when compared with plant during in proliferation stage.

**Fig. 5:**
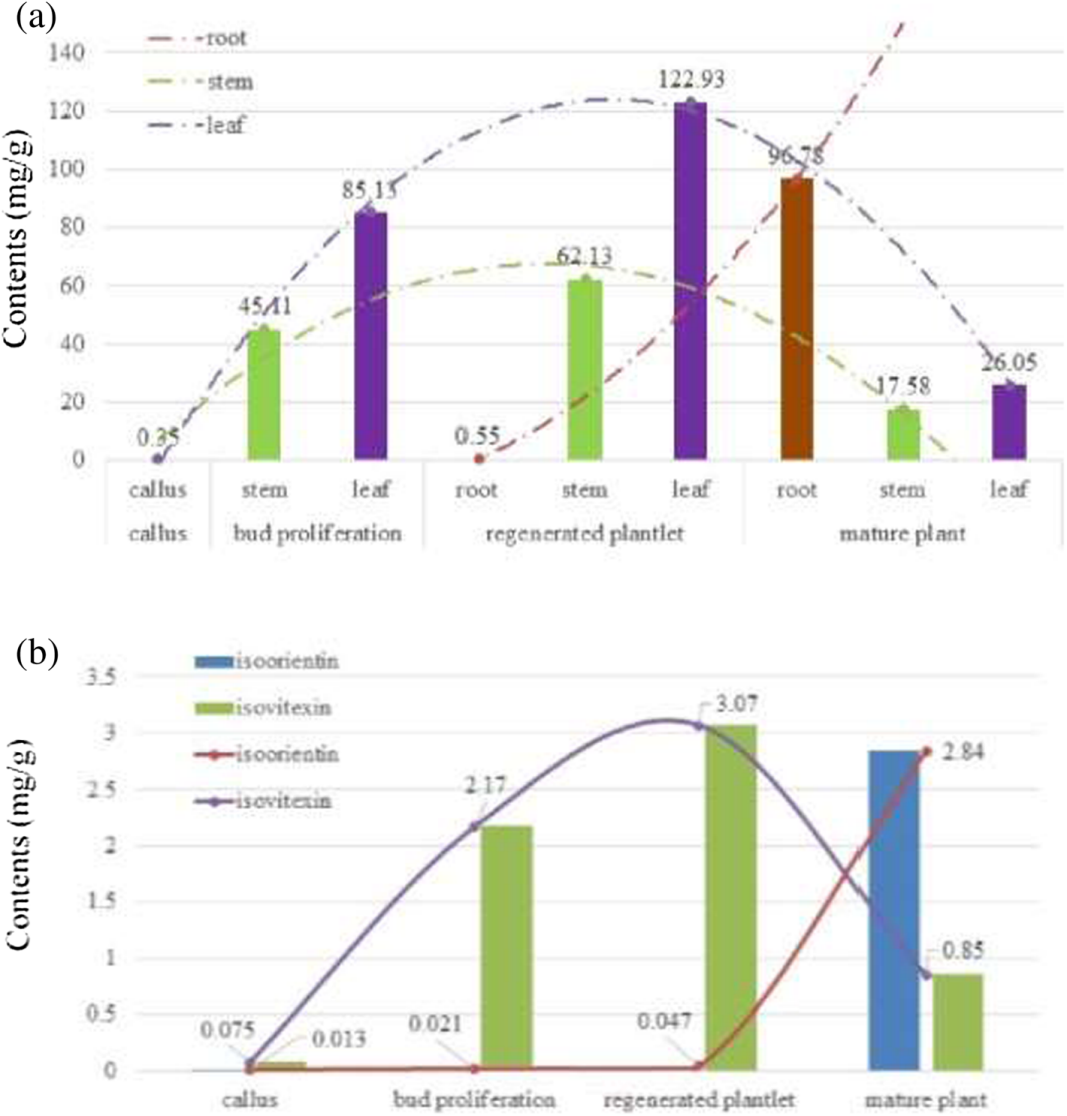
**a**: Determination of gentiopicroside in different sample. **b**: Determination of isoorientin and isovitexin in leaf during different stage

Gentiopicroside is the characteristic compound with the highest amount in *G. rigescens*, which serve as standard for quality control of *G. rigescens* (State Pharmacopoeia Commission [2010]; Wang et al. [2012])*.* Combined with previous study on *Gentiana scabra* (Huang et al. [2014]), another raw materials of Gentianae Radix et Rhizoma, 1.8 higher-fold gentiopicroside content was observed in sample of hair root culture than plants grown in greenhouse. Although leaf in regenerated plantlet contains the highest gentiopicroside yield in this study, it can only be used for industrial extraction of gentiopicroside rather than medical application because the efficacy of herb medicine, to large extend, is derived from synergistic effect of metabolites. The variation on the accumulation of gentiopicroside in different stage is present in Fig. 5a. An interesting phenomenon that gentiopicroside content in root is significant raised with evidently decrease in leaf and stem, when the root start to regenerate. During this stage, root growth rate is far higher than in stem and leaf with the occurrence of gentiopicroside growth, which could be consistent with the results of specific-tissue analysis where the distribution of secondary metabolites vary in different tissue. Moreover, gentiopicroside in aerial parts may translocate into root or transform into other metabolites when the root start to regenerate.

In leaf, a significant negative correlation is found between isoorientin and isovitexin (Fig. 5b). From the biosynthetic pathway of view, it can be explain that these two flavonoids are competitive relationship in a common biosynthetic pathway. Additionally, isovitexin can also be considered as a precursor of isoorientin and *O*-glycosidic isovitexin in this pathway. This may explain why the isovitexin first increase during proliferation stage, and then decrease.

## Conclusions

In the present study, the combination use of FT-IR, LC-UV-MS/MS and chemometrics was designed for investigation of the variation on metabolites during different growing stage of *G. rigescens*. For whole metabolome, the molecular structure-analyte relationships are significantly different between plants during proliferation stage and mature plants according to FT-IR analysis. Combined with LC-UV-MS/MS, mature plants contains more abundant secondary metabolites than plants during proliferation stage, whereas the higher content of some characteristic metabolites like gentiopicroside and peak a are observed in plants during proliferation. Moreover, the distribution and accumulation of metabolites, together with biosynthetic pathway, are associated with plant growth and significantly vary during different growing stage. In practical application, the root in mature plants with rich chemical components could be of better quality for medicinal application, whereas leaf in regenerated plantlet would be used for industrial extraction of gentiopicroside. These results provide evidence for reasonable exploitation and distinct usage of *G. rigescens* during different growing stage.

## Additional file

## Electronic supplementary material

Additional file 1: **Figure S1.** Mass spectrum of isoorientin, isovitexin, peak b and peak c. **Figure S2.** Mass spectrum of peak d-g. **Figure S3.** Mass spectrum of peak a. **Table S1** Fuzz identification of characteristic peaks in UPLC profiling by mass spectrometry. (DOCX 1949 kb) (DOCX 2 MB)

Below are the links to the authors’ original submitted files for images.

Authors’ original file for figure 1

Authors’ original file for figure 2

Authors’ original file for figure 3

Authors’ original file for figure 4

Authors’ original file for figure 5

## Abbreviations

BP: Bud proliferation
FT-IR: Fourier transform infrared spectroscopy
LOD: The limits of detection
LOQ: The limits of quantification
LC-MS/MS: Liquid chromatography tandem mass spectrometry
MP: Mature plant
MRM: Multiple reaction monitoring
RP: Regenerated plantlet
