# Supplementary material for: Investigation of metabolites accumulation in medical plant Gentiana rigescens during different growing stage using LC-MS/MS and FT-IR
Source: Bot Stud. 2015 May 27;56:14. doi: 10.1186/s40529-015-0094-6 (PMC5434671; doi:10.1186/s40529-015-0094-6)
Supplement: Supplementary file 1 — Additional file 1: Figure S1. Mass spectrum of isoorientin, isovitexin, peak b and peak c. Figure S2. Mass spectrum of peak d-g. Figure S3. Mass spectrum of peak a. Table S1 Fuzz identification of characteristic peaks in UPLC profiling by mass spectrometry. (DOCX 1949 kb) (DOCX 2 MB) [file 40529_2015_94_MOESM1_ESM.docx]

**isoorientin**

**
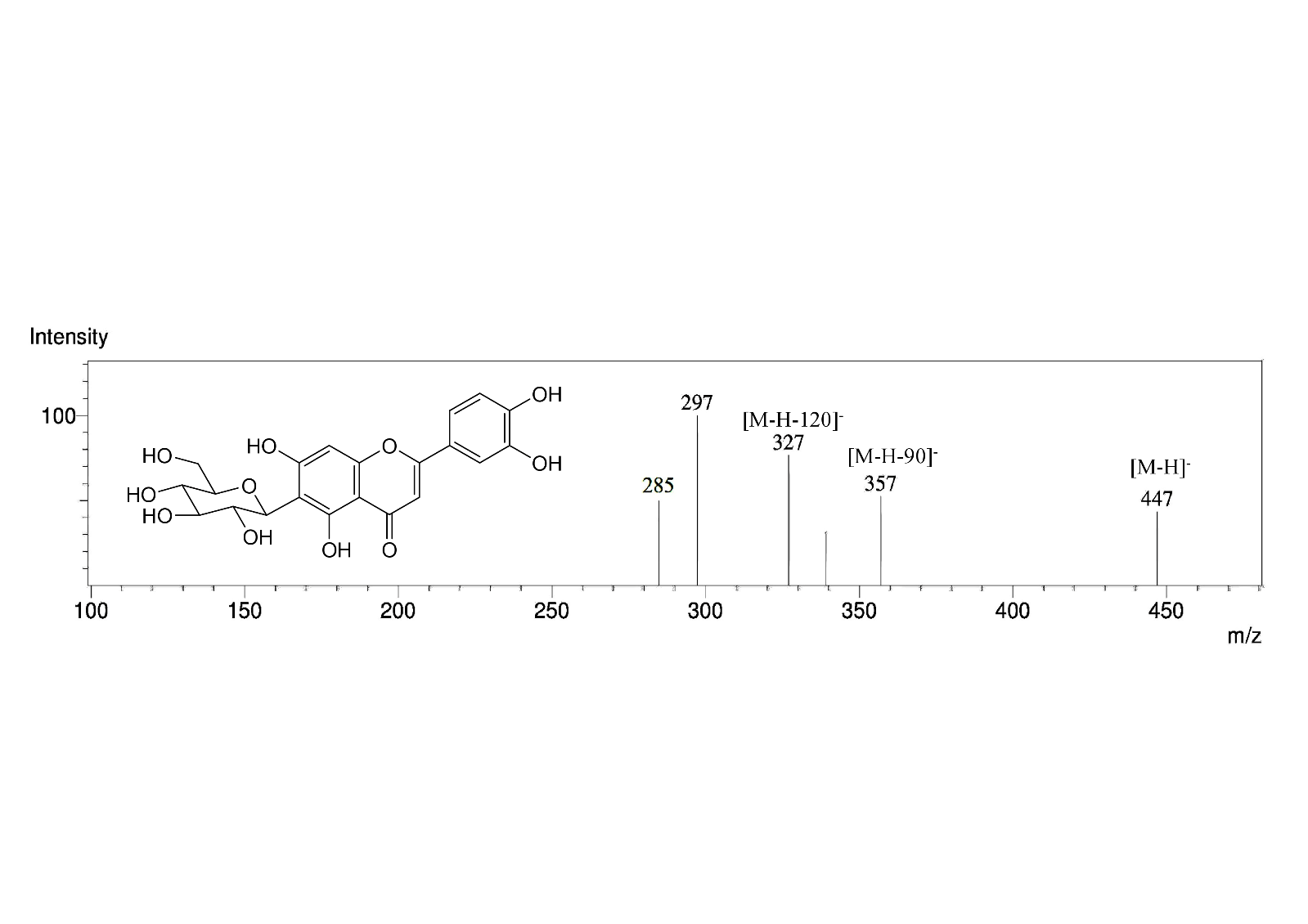
**

**isovitexin**


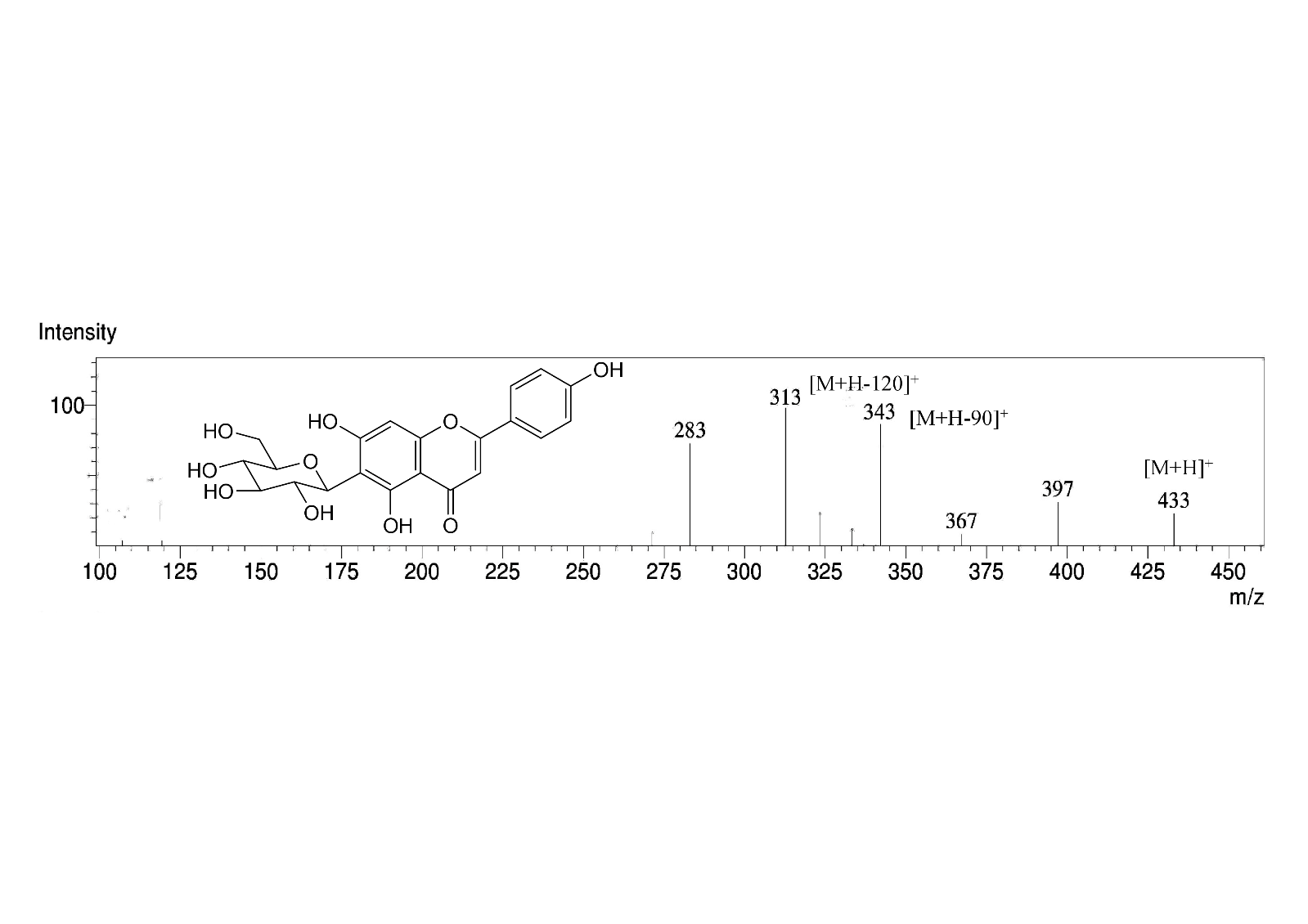


**peak b**


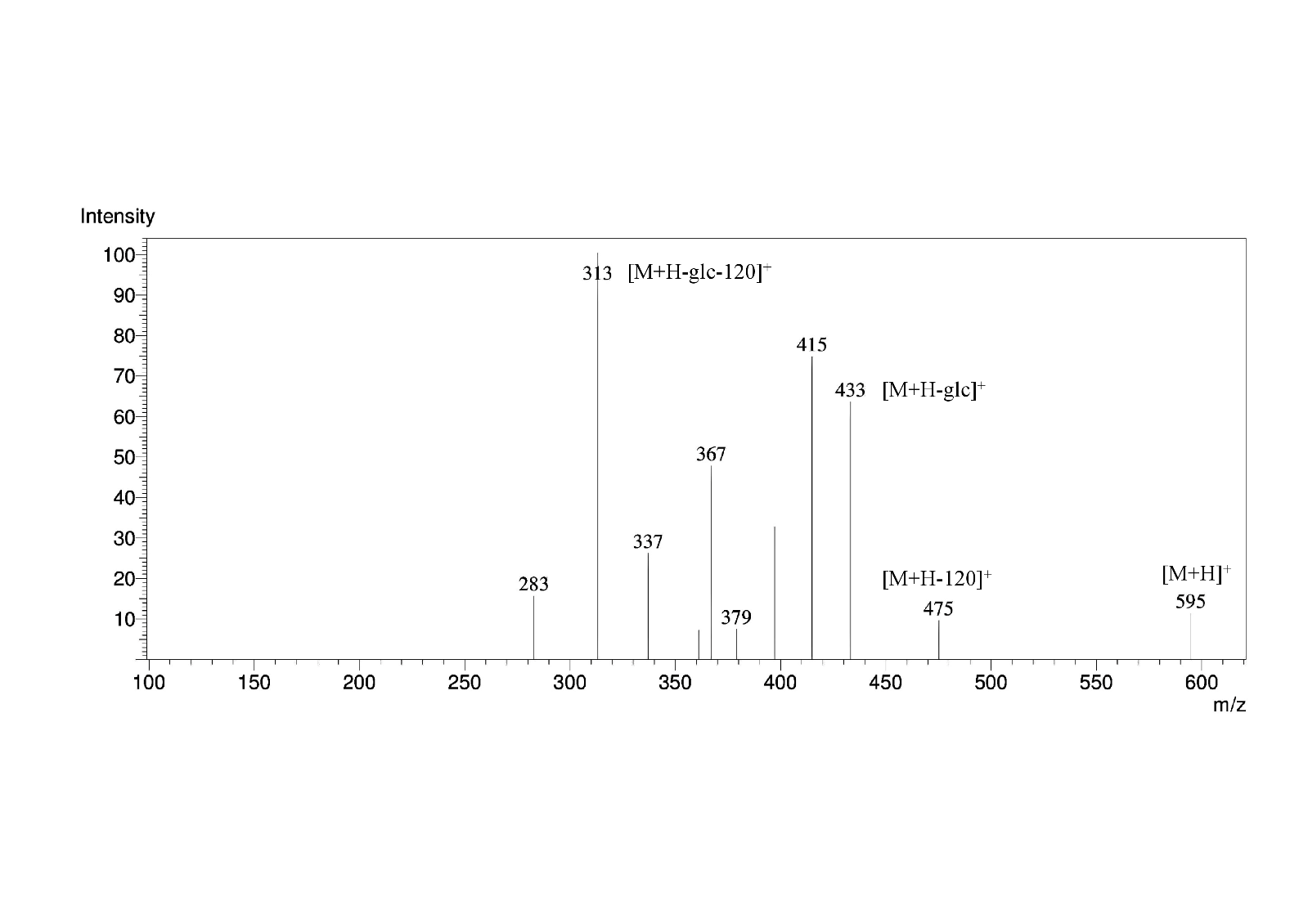


**peak c**


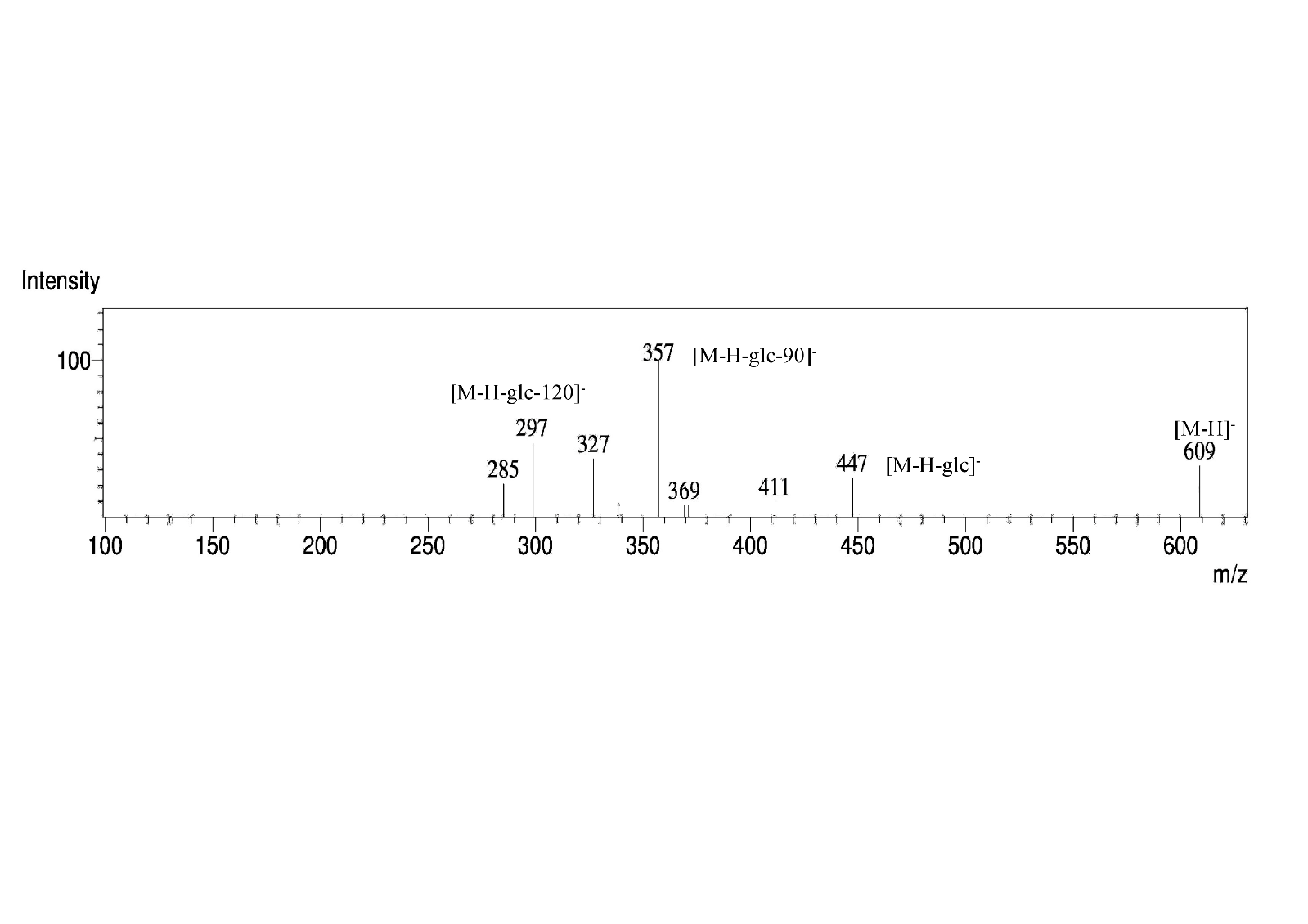


**Figure S1.** Mass spectra of isoorientin, isovitexin, peak b and peak c

**Peak d** (2′-(2,3-dihydroxybenzoyl)-gentiopicroside)

**
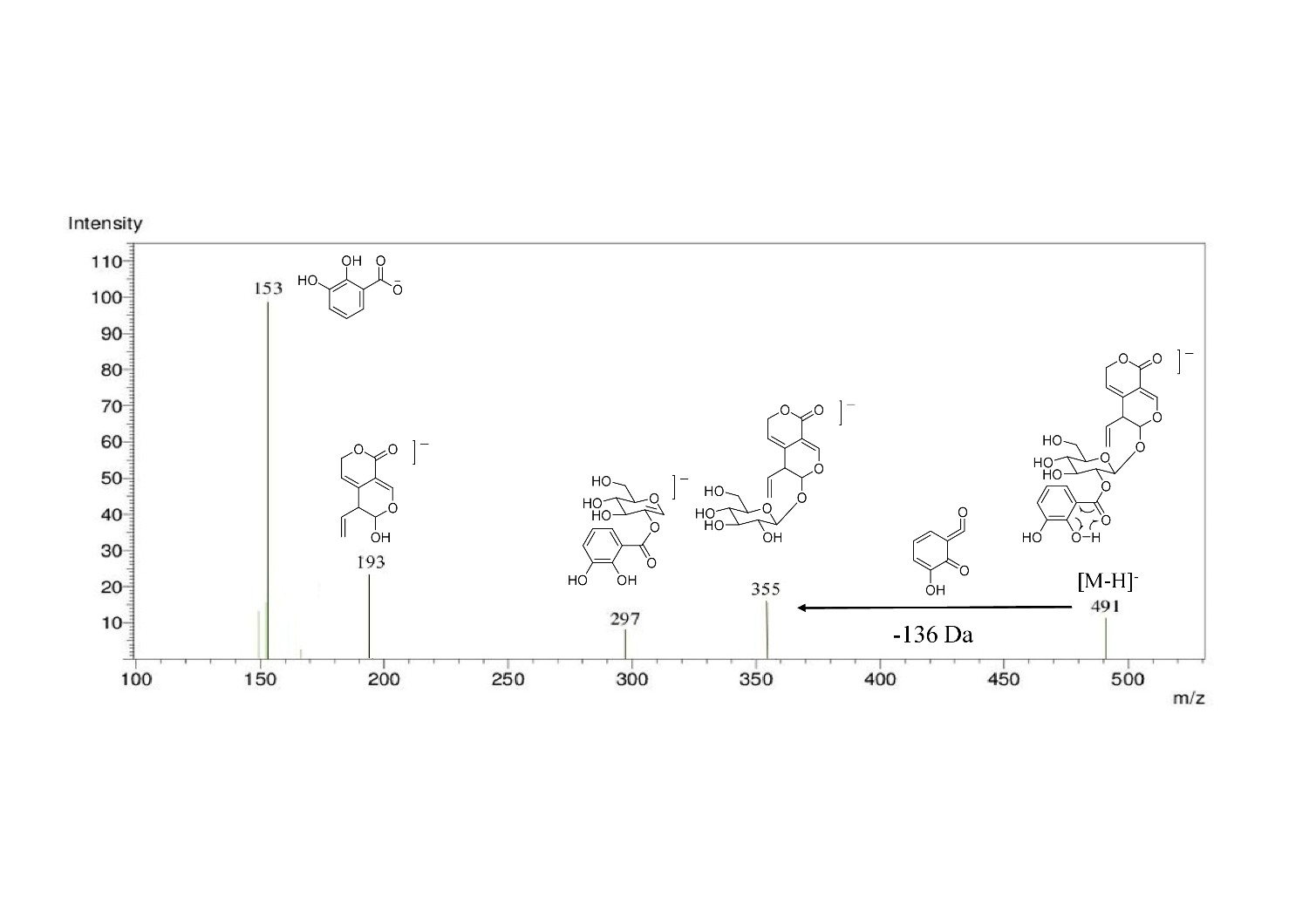
**

**Peak e (**2′-(2,3-dihydroxybenzoyl)-sweroside)

**
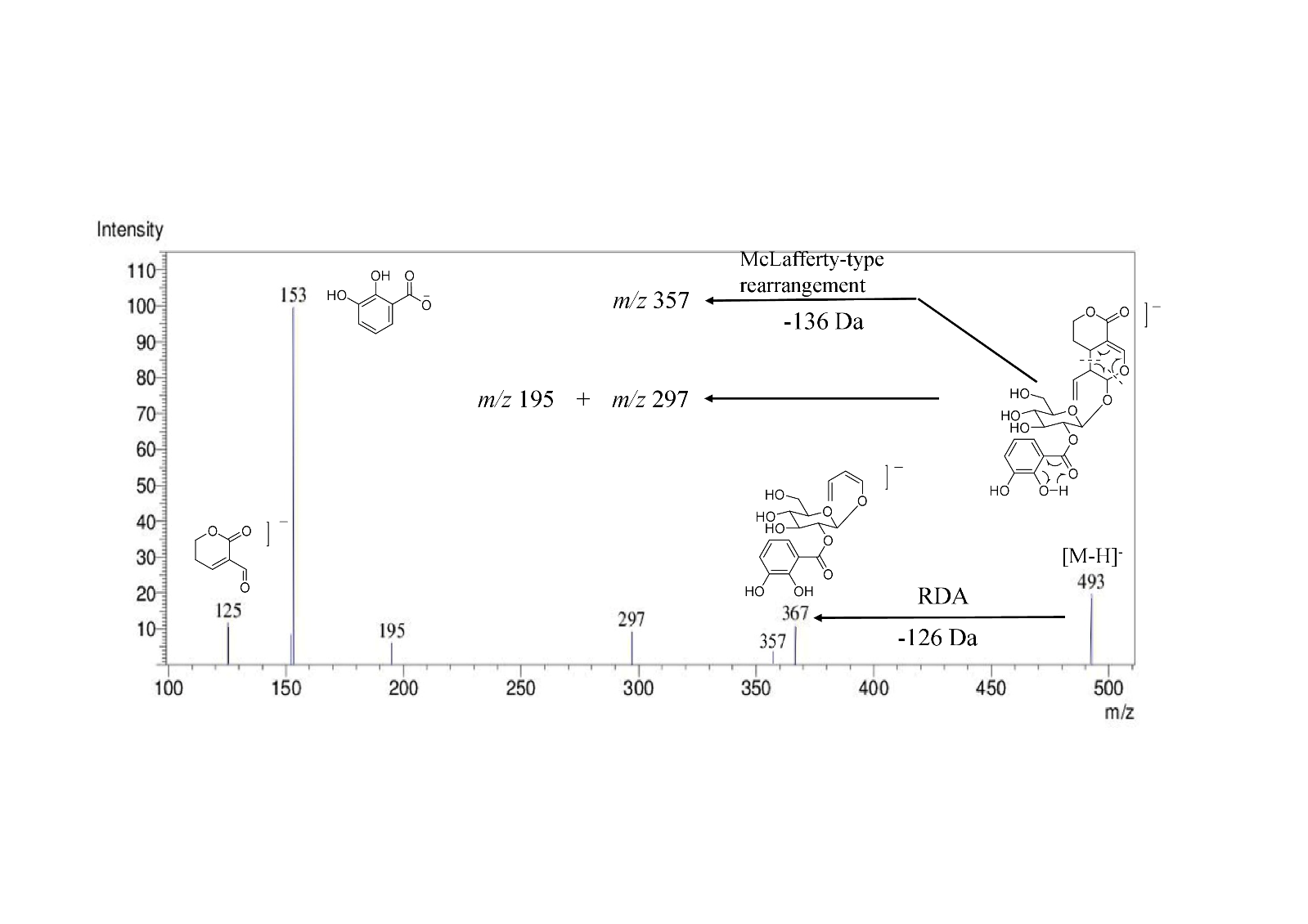
**

**Peak f (**macrophyllosides A)

**
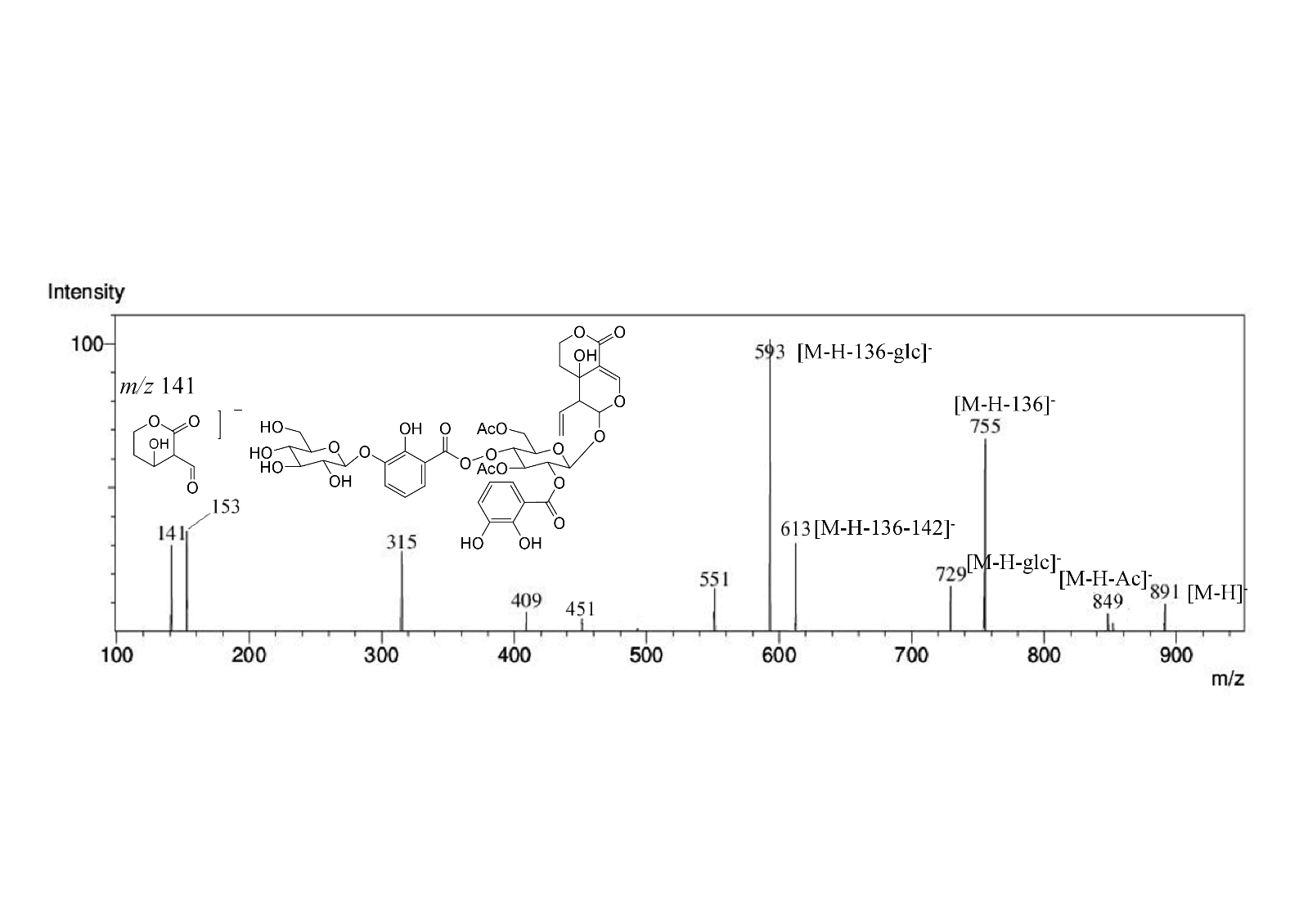
**

**Peak g (**macrophyllosides B)

**
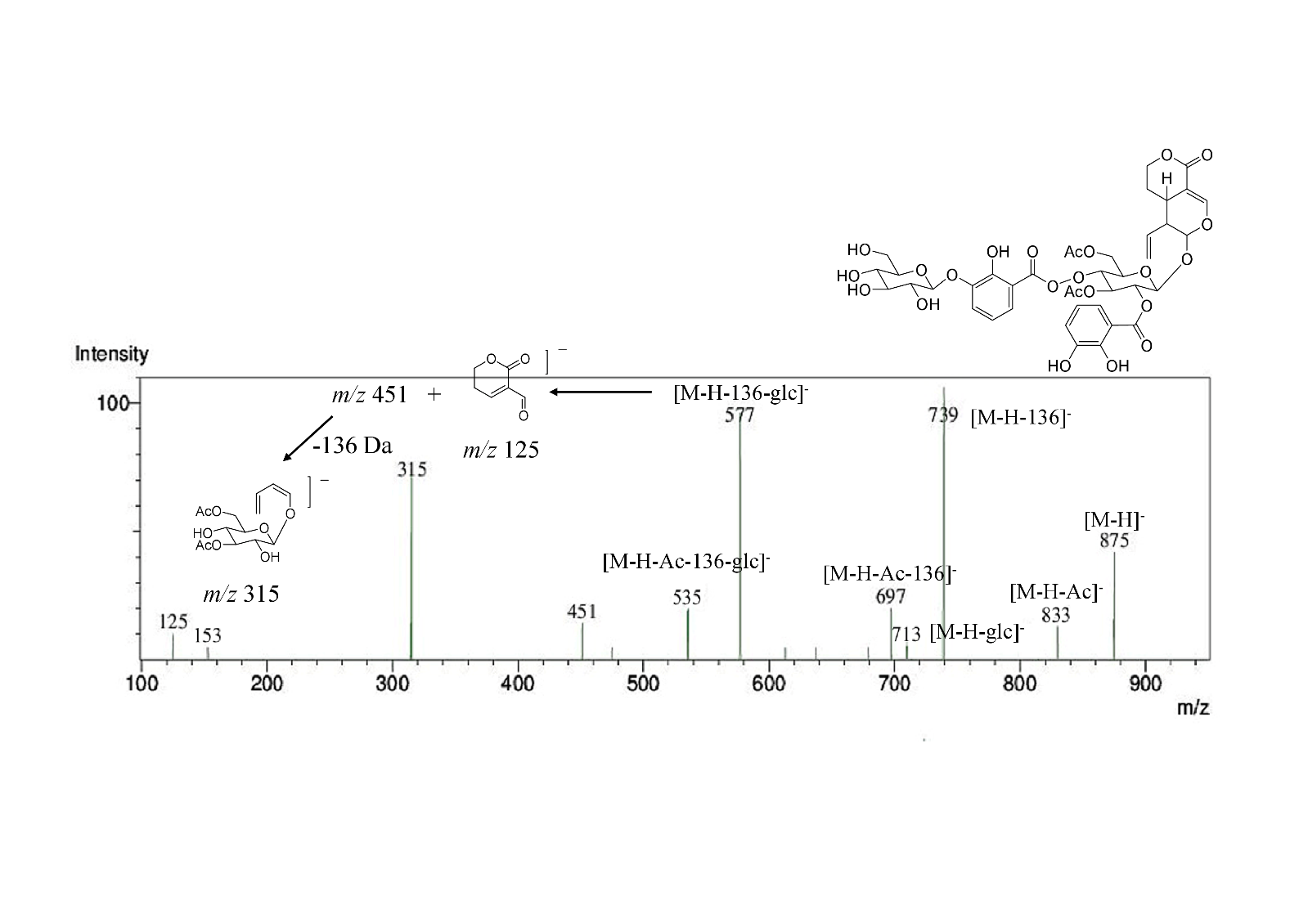
**

**Figure S2.** Mass spectra of peak d-g

**Peak a (**2-hydroxy-3-glucopyranosyl-benzoic acid)

**
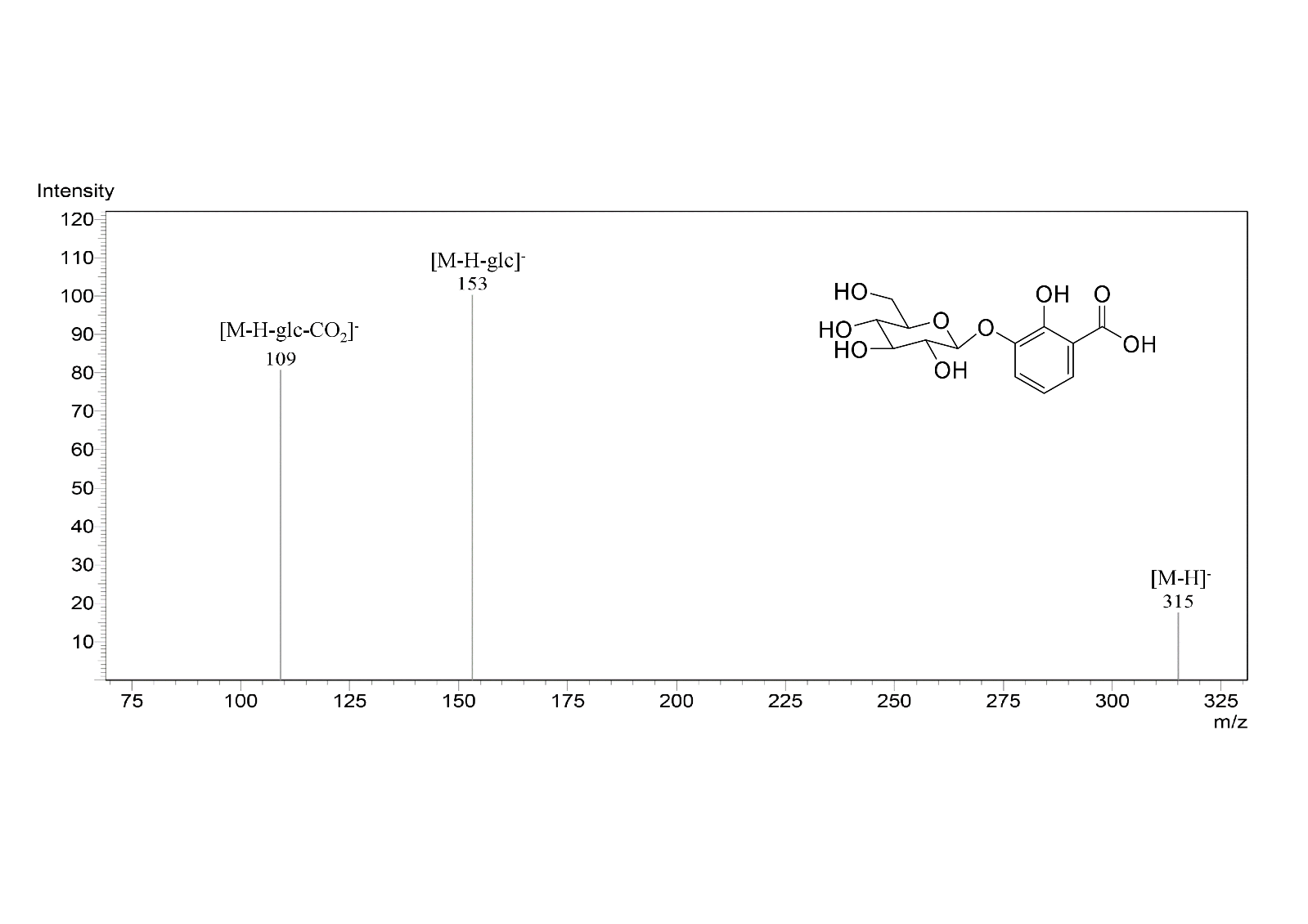
**

**Figure S3.** Mass spectrum of peak a

Table S1 Fuzz identification of characteristic peaks in UPLC profiling by mass spectrometry

| No. | Rt (min) | Measured (*m/z*) | Calculated (*m/z*) | Error (ppm) | Formula | Identity | Ref |
| --- | --- | --- | --- | --- | --- | --- | --- |
| a | 1.67 | 315.0832 [M-H]^-^ | 315.0812 | -2.73 | C_13_H_16_O_9_ | 2-hydroxy-3-glucopyranosyl-benzoic acid | Xu *et al.,* (2009a) |
| b | 2.55 | 593.1513 [M-H]^-^ | 593.1512 | -0.43 | C_27_H_30_O_15_ | Isovitexin-*O*-glucoside | - |
| c | 3.9 | 611.1503 [M-H]^-^ | 609.1521 | 2.37 | C_27_H_30_O_16_ | Isoorientin-*O*-glucoside | - |
| d | 4.9 | 491.1180 [M-H]^-^ | 491.1195 | 1.83 | C_23_H_24_O_12_ | 2′-(2,3-dihydroxybenzoyl)-gentiopicroside | Suyama *et al.,* (2013) |
| e | 5.2 | 493.1382 [M-H]^-^ | 493.1351 | -3.12 | C_23_H_26_O_12_ | 2′-(2,3-dihydroxybenzoyl)-sweroside | Suyama *et al.,* (2013) |
| f | 5.7 | 875.2246 [M-H]^-^ | 875.2251 | 1.29 | C_40_H_44_O_22_ | macrophyllosides A | Tan *et al.,* (1996) |
| g | 6.7 | 891.2233 [M-H]^-^ | 891.2201 | -3.44 | C_40_H_44_O_23_ | macrophyllosides B | Tan *et al.,* (1996) |
