# Supplementary figures and images for: Investigation of metabolites accumulation in medical plant Gentiana rigescens during different growing stage using LC-MS/MS and FT-IR
Source: Bot Stud. 2015 May 27;56:14. doi: 10.1186/s40529-015-0094-6 (PMC5434671; doi:10.1186/s40529-015-0094-6)

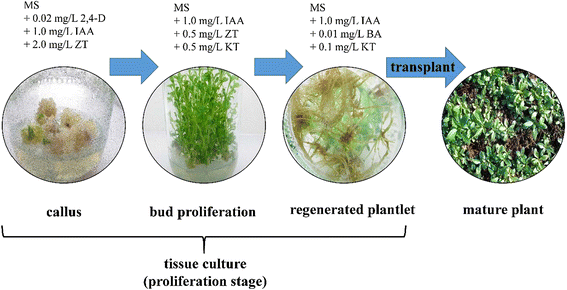

Supplement: Supplementary file 2 — Authors’ original file for figure 1 [file 40529_2015_94_MOESM2_ESM.gif]

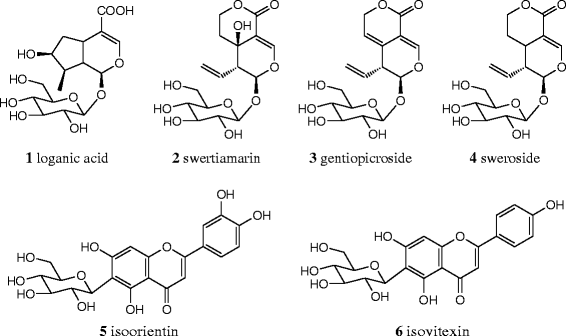

Supplement: Supplementary file 3 — Authors’ original file for figure 2 [file 40529_2015_94_MOESM3_ESM.gif]

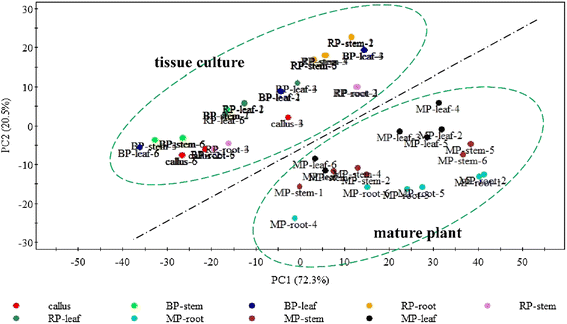

Supplement: Supplementary file 4 — Authors’ original file for figure 3 [file 40529_2015_94_MOESM4_ESM.gif]

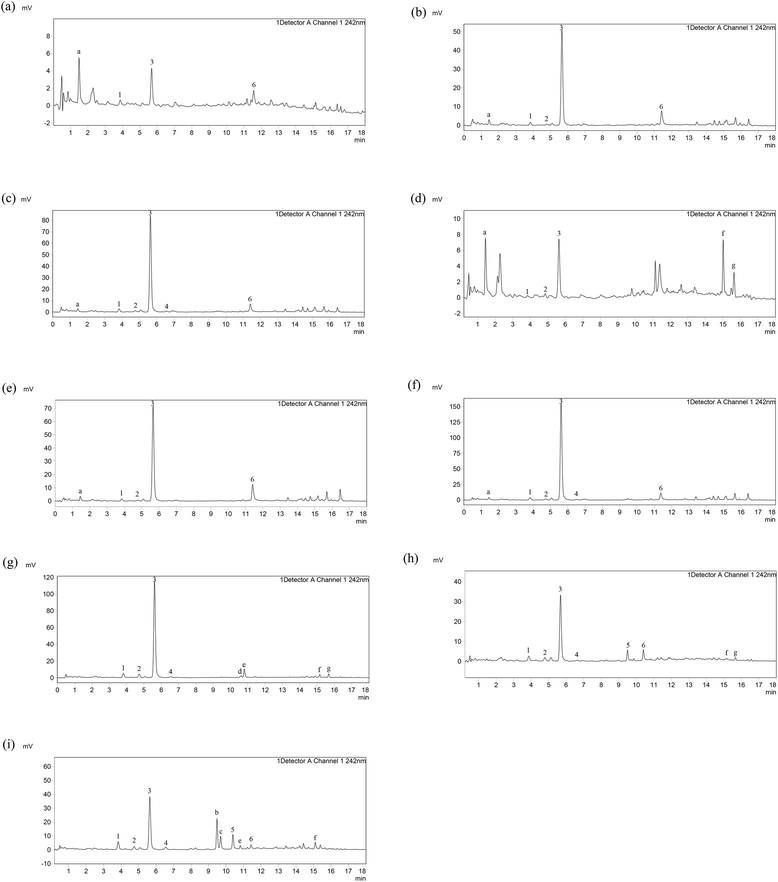

Supplement: Supplementary file 5 — Authors’ original file for figure 4 [file 40529_2015_94_MOESM5_ESM.gif]

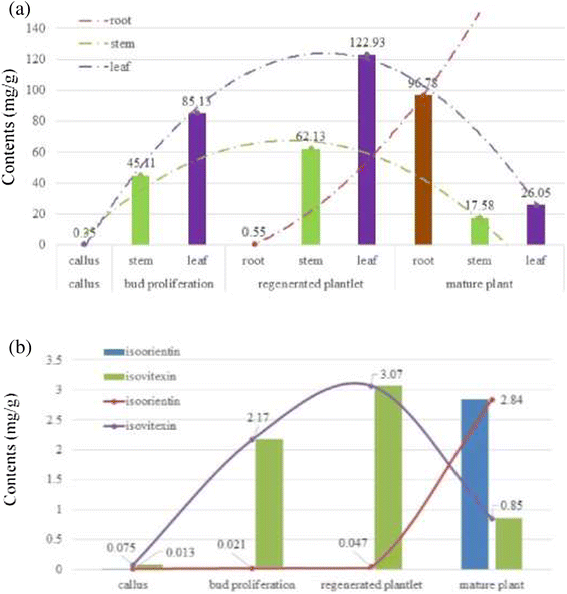

Supplement: Supplementary file 6 — Authors’ original file for figure 5 [file 40529_2015_94_MOESM6_ESM.gif]
